# COVID-19-associated mucormycosis: the rise and fall of an epidemic within a pandemic - a systematic review of 1,482 cases (2019-2025)

**DOI:** 10.1590/0037-8682-0504-2025

**Published:** 2026-07-17

**Authors:** Júlia Carvalho Mendes, Raphaella Vieira Lima, Rafaela Moreira da Silva, Diego Batista Carneiro de Oliveira, Kennio Ferreira-Paim, Fernanda Machado Fonseca

**Affiliations:** 1 Universidade Federal do Triângulo Mineiro, Departamento de Biomedicina, Uberaba, MG, Brasil.; 2 Universidade Federal do Triângulo Mineiro, Departamento de Microbiologia, Imunologia e Parasitologia, Uberaba, MG, Brasil.

**Keywords:** Mucormycosis, COVID-19, Diabetes mellitus, Corticosteroids, Vaccination, Mucorales

## Abstract

During the COVID-19 pandemic, an unprecedented outbreak of mucormycosis affected tens of thousands of patients, mainly in India. This study reviewed the available published cases up to 2022 and updated the epidemiological curve up to November 2025. PRISMA-guided systematic review of PubMed, Embase, and SciELO (Dec. 2019-Dec. 2022), supplemented by official reports and cohort studies published until 2025. We analyzed 1,482 cases from 39 studies (90.8% conducted in India; 69.7% male individuals; mean age 52 years). Diabetes mellitus was present in >78% of patients, and 64.3% received corticosteroids. Rhino-orbital (40.7%) and rhino-orbito-cerebral (33.9%) were the dominant forms of mucormycosis. Amphotericin B was used in 49.5% of cases, and surgical debridement in 55.5%. The mortality rate was 25%. After 2022, the incidence collapsed globally: India dropped from >47,000 cases in 2021 to <100 cases/year; Europe, the Middle East, and Latin America reported zero outbreaks in 2024-2025. COVID-19-associated mucormycosis represented a major opportunistic infection during the acute phase of the pandemic. Although its incidence declined markedly after widespread vaccination implementation, improved corticosteroid use, and better glycemic control, it remains a serious and life-threatening condition in vulnerable populations. The findings highlight important risk factors, clinical challenges, and management considerations that remain relevant to inform current clinical practice and future post-pandemic scenarios.

## INTRODUCTION

In December 2019, the first reports emerged of a group of patients presenting with respiratory infection caused by a previously unidentified microbial agent in Wuhan, China. These cases were later associated with the novel coronavirus (COVID-19), named Severe Acute Respiratory Syndrome Coronavirus 2 (SARS-CoV-2)^1^. COVID-19 cases quickly spread from China to several countries, causing a pandemic and raising global health concerns^2^.

Phylogenetic analyses identified SARS-CoV-2 as a member of the Coronaviridae family, recognizing it as the seventh species known to infect humans among six others previously identified^3^. Genomic studies also classified the virus as a Betacoronavirus due to its origin in mammals, such as bats. It is an enveloped virus with a single-stranded RNA genome and a high mutation capacity^4^.

Infected individuals often present with symptoms such as malaise, dyspnea, persistent cough, and fever. Pneumonia develops in more severe cases^5^. When the virus infects alveolar cells and the immune system does not respond effectively, a cytokine imbalance occurs, with a reduction in circulating lymphocytes, leading to significant pulmonary endothelial damage^6^. In severe cases, patients may develop acute respiratory distress syndrome (ARDS), which frequently requires assisted mechanical ventilation^7^.

Critically ill patients with COVID-19 often experience profound immunosuppression, increasing susceptibility to secondary infections^8^, which may explain the numerous reports of invasive fungal co-infections since the onset of the pandemic^7^.

Invasive fungal infections spread through internal organs, such as the lungs and central nervous system (CNS), among others, through the bloodstream. They predominantly affect immunocompromised and hospitalized patients^9^. When these infections are established in SARS-CoV-2-infected individuals, they can rapidly progress to death, especially in patients who require prolonged admission to the intensive care unit (ICU)^10^. One such highly invasive opportunistic infection increasingly reported in association with COVID-19 is mucormycosis. During the pandemic, several cases of severe patients with COVID-19 co-infected with fungi of the order Mucorales^11^ were reported. In India, the mucormycosis outbreak associated with SARS-CoV-2 was an epidemic within the pandemic^12^.

*Mucor* spp*.* and *Rhizopus* spp*.* are mucormycetes commonly found in soil, plants, wood, decaying fruits and vegetables, and even in the air. Immunocompetent individuals exposed to these spores usually do not develop disease due to the effective phagocytic action of respiratory tract macrophages^13^. However, in immunocompromised patients, mucormycosis can be fatal, potentially affecting the nasal and paranasal sinuses, nose, CNS, lungs, gastrointestinal tract, jaw bones, and even the optic nerve^11^ ^,^ ^14^. The invasion of vascular endothelial cells and subsequent ischemia and necrosis associated with these infections darken the tissue, leading to the popular reference as black fungus^14^.

COVID-19 patients co-infected with Mucorales often exhibit metabolic changes favorable to fungal germination. Viral infection induces metabolic acidosis, impairs pulmonary function, and reduces oxygenation. Hyperglycemia occurs commonly as a consequence of the diabetogenic effects COVID-19, excessive protein glycosylation (including transferrin and ferritin), elevated serum iron and reduced macrophage function^15^. Additionally, T-cell depletion facilitates spore germination and hyphal formation, as T cells generally inhibit fungal proliferation under physiological conditions^16^.

Mucorales fungi acquire iron from the host, facilitating fungal proliferation^17^. SARS-CoV-2 infection has been associated with alterations in iron metabolism and hyperferritinemia; however, the exact mechanisms that link these changes to fungal proliferation remain unknown. Furthermore, COVID-19 treatment often involves glucocorticoids, which elevate blood glucose through proteolysis and glycogenolysis and suppress immune function by decreasing immune cell activation^18^. Uncontrolled diabetes mellitus is a well-established risk factor for COVID-19/mucormycosis co-infection. Elevated blood glucose weakens immune responses and promotes the germination of fungal spores by activating the endothelial growth factor receptor pathway, allowing fungal invasion^19^.

Although the incidence of COVID-19-associated mucormycosis has declined after widespread vaccination and improved preventive strategies, infection remains a significant clinical concern. The COVID-19 pandemic has highlighted important risk factors, diagnostic challenges, and therapeutic limitations that extend beyond the context of the pandemic. Therefore, a comprehensive understanding of mucormycosis in this setting remains essential to inform current clinical practice and guide future research priorities.

Given the growing association between COVID-19 and opportunistic fungal infections such as mucormycosis, this study aimed to provide a comprehensive synthesis of the available evidence on the clinical, epidemiological, microbiological, diagnostic and therapeutic characteristics of COVID-19-associated mucormycosis. Furthermore, it aims to identify key risk factors, highlight existing knowledge gaps, and discuss the implications of these findings for current clinical practice and future post-pandemic scenarios.

## METHODS

A systematic literature search was conducted in the PubMed, Embase, and SciELO databases covering the period from December 2019 to December 2022. The search strategy included a combination of controlled vocabulary (MeSH/Entree) and free-text terms applied to titles and abstracts that combine terms related to COVID-19 (COVID-19 or SARS-CoV-2), terms related to mucormycosis (mucormycosis), and specific genera (e.g., Mucor and Rhizopus), as well as the descriptor COVID-19-associated mucormycosis, to maximize sensitivity. The complete and reproducible search strategies for each database, including all search terms, Boolean operators, and field specifications (title/abstract and controlled vocabulary), are provided in the Supplementary Material (Table S1). The search was performed in January 2023.

We applied the PRISMA (Preferred Reporting Items for Systematic Reviews and Meta-Analyses) methodology to guide and organize the research stages^20^. Eligibility criteria were defined a priori, including study design, population, outcomes of interest, and language, as well as study relevance to COVID-19-associated mucormycosis, availability of clinical and/or epidemiological data, and publication as original peer-reviewed studies, case reports, or case series. The included studies were selected by title and abstract selection, followed by full-text review. The eligible articles were written in English and included original peer-reviewed studies, case reports, and case series describing mucormycosis in COVID-19 patients. The exclusion criteria were non-original studies, such as reviews, systematic reviews, meta-analyses, or editorials without original data, as well as studies without full-text availability (e.g., conference abstracts or summaries). Next, Microsoft Excel spreadsheets were used to organize the data extracted from the selected articles. Each study was manually reviewed and added to the database. Using conditional formatting, duplicate studies were identified using the “highlight cell rules” and “duplicate values" functions and then manually excluded.

Two independent reviewers assessed the eligibility of each study based on the title and abstract to ensure the relevance of the included literature. The full texts of the studies were then thoroughly analyzed and only those that met the inclusion criteria were incorporated into the qualitative synthesis.

To describe the temporal evolution of COVID-19-associated mucormycosis beyond the systematic review period, additional data from official epidemiological surveillance reports and large observational studies (non-systematically collected) published between 2023 and early 2025 were included. These data were analyzed separately to characterize post-pandemic trends. To minimize duplication bias, studies were screened for overlapping populations using predefined criteria based on geographic location, study period, and institutional setting. When potential overlap was identified, only the most comprehensive data set was included. 

The methodological quality of included studies was assessed using the Joanna Briggs Institute (JBI) Critical Assessment Checklists^21^ for case reports and case series (Supplementary Table S2 and Supplementary Table S3). Two independent reviewers conducted the evaluation and disagreements were resolved by consensus. Quality assessment was used descriptively and not as a basis for exclusion from the study.

Due to the heterogeneity of study designs and variability in data reporting, only descriptive analyses were performed. No meta-analysis or weighted estimates were conducted.

## RESULTS

**FIGURE 1:**
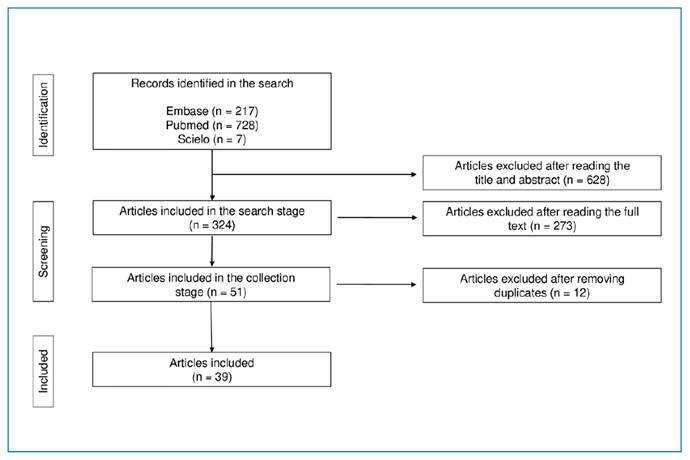
PRISMA flow diagram describing the stages of article selection for this review.

The selected databases initially yielded 952 scientific articles, with 728 (76.5%) in PubMed, 217 (22.8%) in Embase, and 7 (0.73%) in SciELO. After title and abstract screening, 324 articles remained. Following the full-text analysis, 51 articles were selected. After removing duplicates, the final review consisted of 39 studies (Figure 1).

The 39 included studies reported 1,482 cases of COVID-19-associated mucormycosis. Most of the cases occurred in India (1,345; 90.8%), followed by Egypt with 78 cases (5.3%), and Iran with 39 cases (2.7%). Other countries contributed fewer than 1% of cases individually (approximately n = 13.4 cases). Regarding sex distribution, 1,032 patients (69.7%) were male and 450 (30.3%) were female. The ages of patients ranged from 41 to 73 years, with a mean of 52 years among the 10 analyzed case reports. The etiological agent was identified in 383 (25.8%) cases. Among them, *Mucor* spp. was isolated in 265 cases (69.2%) and *Rhizopus* spp. in 118 cases (30.8%). In 1,099 (74.2%) cases, only the fungal order (*Mucorales*) was identified without specifying the genus or species.

The primary clinical forms of mucormycosis were rhino-orbital (603 cases; 40.7%) and rhino-orbito-cerebral (503 cases; 33.9%). Rhino-sinus (159 cases; 10.7%), rhino-sino-orbital (85 cases; 5.7%), and rhino-cerebral (72 cases; 4.9%) were additional presentations, as well as less frequent forms, such as gastrointestinal, palatal, cutaneous, mandibular/maxillary, pulmonary, orbital, renal, disseminated, and subglottic involvement (59 cases; 4%). Corresponding to these clinical forms, the predominant symptoms in COVID-19/mucormycosis co-infected patients were fever, headache, ptosis, proptosis, ophthalmoplegia, facial or ocular edema, and erythema. Less common manifestations included melena, abdominal pain, ulcers, toothache, and jaw pain.

The most prevalent comorbidity was diabetes mellitus, followed by hypertension, kidney disease, cardiovascular disorders, liver disease, and chronic pulmonary disease. Many patients also presented multiple comorbidities simultaneously. Among the 1,482 patients analyzed, 953 (64.3%) received or were undergoing corticosteroid therapy (e.g., methylprednisolone or dexamethasone) for COVID-19 (Table 1).

Oxygen therapy was required for 352 patients (23.7%), whereas invasive mechanical ventilation was necessary for 140 (9.4%). One patient (0.07%) required both oxygen modalities, and 28 cases (1.89%) did not need ventilatory support.

**TABLE 1:** Clinical forms of mucormycosis according to the number of affected patients.

Type of involvement	Number of patients (%)
Mandibular/Maxillary	9 (0.6)
Orbital	2 (0.13)
Pulmonary	31 (2.1)
Renal/Disseminated	7 (0.48)
Rhino-Orbital	603 (40.7)
Rhino-Cerebral	72 (4.86)
Rhino-Sinus	159 (10.7)
Rhino-Sinus-Orbital	85 (5.73)
Rhino-Orbito-Cerebral	503 (33.9)
Subglottic	1 (0.07)
Cutaneous/Palatal/Gastrointestinal	10 (0.67)
**Total**	**1,482 (100)**

Antifungal therapy included amphotericin B (liposomal, deoxycholate, or lipid complex), used in 733 patients (49.5%). Posaconazole was used alone in 78 patients (5.3%), isavuconazole in 19 (1.3%), and a combination therapy with liposomal amphotericin B plus posaconazole, caspofungin, or isavuconazole was applied to 68 patients (4.6%). Treatment data were unavailable in 584 cases (39.4%). Surgical intervention was required in 822 cases (55.5%). Among the total co-infected patients, 370 (25%) died, 960 (64.8%) recovered clinically, and 152 (10.3%) had no reported outcome (Table 2). 

**TABLE 2:** Clinical features, comorbidities, and mortality of patients with COVID-19-associated mucormycosis.

Author	Year	Country	Type of study	N.of patients	Age (years)	Sex	Comorbidities	Form of mucormycosis	Steroid	OX/VM	Treatment	SI	Death
AGGARWAL SK., et al.^47^	2021	IND	CA	13	Mean 51,5	M(10) F(3)	DM+HT (1)	RO (13)	Yes (12)	OX (3)	-	Yes (13)	Yes (2)
							DM+H (6)						
							DM (5)						
							DM+Dl (1)						
AHMED OF., et al.^48^	2022	IRQ	MCS	4	Mean 59,2	F(4)	DM (1)	Pulmonary (4)	Yes (4)	MV (2)	LAMB (4)	Yes (4)	Yes (1)
							DM+HD (1)						
ALEKSEYEV K., et al.^49^	2021	USA	CA	1	41	M	DM	RC	Yes	No	LAMB	Yes	No
ALLOUSH TK., et al.^50^	2022	EGY	RE	14	Mean 64,7	M(8) F (6)	DM (13)	ROC (14)	Yes (14)	OX (8)	LAMB (10)	Yes (9)	Yes (3)
							H (1)				POSA (4)		
							HD (2)						
							N (1)						
AMIRZARGAR B., et al.^51^	2022	IRN	CA	1	51	F	DM+HT	S	Yes	No	LAMB	Yes	No
ARORA R., et al^52^.	2021	IND	CS	60	Mean 57	M(45) F(15)	DM (49)	ROC (60)	Yes (60)	OX (30)	LAMB (60)	Yes (10)	No (60)
ARORA U., et al^53^.	2022	IND	CC	152	Mean 48,2	M(105) F(47)	DM (145)	RS (44)	Yes (100)	OX (53)	-	-	-
							NP (6)	RO (72)					
							T (2)	ROC ( 22)					
								RSO (8)					
								Pulmonary (2)					
								D (1)					
								OR (2)					
								CT (1)					
AVATEF FAZELI, M., et al.^54^	2021	IRN	OS	12	Mean 62,1	M(5) F (7)	DM (2) DM+H (4) DM+OD (6)	RO (12)	Yes (8)	-	LAMB (12)	Yes (12)	Yes (8)
BARMAN ROY D., et al.^55^	2021	IND	CA	5	Mean 49,6	M(3) F (2)	DM (1)	RO (5)	Yes (4)	No (5)	LAMB (4)	Yes (5)	No (5)
							DM+H (1)		No (1)		LAMB + POSA (1)		
							DM+H+HT (1)						
							NP (1)						
BAYRAM N., et al.^56^	2021	TUR	OS	11	Mean 73,1	M(9) F(2)	DM+ H (8)	RO (n=11)	Yes (11)	-	LAMB (11)	Yes (11)	Yes (7)
							NP (5)						
							MS (1)						
BHADANIA S., et al.^57^	2021	IND	CS	62	Mean 53	M(41) F(21)	DM (28)	RS (31) ROC (31)	-	-	-	-	-
							DM+H (16)						
							H (4)						
BHANUPRASAD K., et al.^58^	2021	IND	AC	132	Mean 50,5	M(101) F(31)	DM (129)	RO (93) RC (39)	Yes (73)	OX (19)	-	-	Yes (13)
							HD (5)						
							LGD (3)						
							NP (7)						
CHAKRAVARTYJ., et al.^59^	2022	IND	OS	208	Mean 52,8	M(136) F(72)	DM (200)	ROC (204)	Yes (131)	OX (79)	AmB (208)	Yes (189)	Yes (68)
							H (61)	GI (1)					
							NP (8)	Pulmonary (3)					
CHANG CY., et al.^60^	2022	MYS	CA	1	42	M(1)	DM	RO	Yes	OX	AmBD	Yes	No
DRAVID A., et al.^61^	2022	IND	RE	59	Median 52	M(47) F(12)	DM (36)	ROC (58) R (1)	Yes (59)	MV (7)	LAMB (3)	Yes (56)	Yes (15)
							H (24)			OX (4)	LAMB + POSA (56)		
							NP (9)						
							HD (7)						
							LD (2)						
ESWARAN S., et al.^62^	2021	IND	CA	1	31	M	HG	ROC	Yes	No	LAMB	Yes	No
FARGHLY YOUSSIF S., et al.^63^	2022	EGY	CS	33	Mean 58	M(21) F(12)	DM (21) H (15) NP (3) LD (4) HD (3)	ROC (32) RO (1)	Yes (33)	-	LAMB (10)	Yes (3)	Yes (7)
FOUAD YA., et al.^65^	2021a	EGY	OS	5	Mean 51,8	M(4) F(1)	DM (3) DM+NP (2)	ROC (2) RO (3)	Yes (5)	MV (1)	LAMB (5)	Yes (2)	Yes (3)
FOUAD YA., et al.^64^	2021b	EGY	MCS	26	Mean 62,5	M(14) F(12)	DM (11)	RO (26)	Yes (20)	-	LAMB (26)	Yes (17)	Yes (12)
							H (7)						
							NP (3)						
							HD (2)						
							Asthma (2)						
							LD (1)						
GUPTA R., et al.^66^	2021	IND	OS	115	Mean 54,2	M(81) F (34)	DM (98)	RO (63)	Yes (115)	OX (78)	-	-	Yes (25)
							HD (18)	ROC (25)		MV (13			
							NP (12)	RS (27)		)			
HASHEMINASAB M., et al.^67^	2022	IRN	CA	8	Mean 51,8	M(6) F(2)	DM (8)	MX (8)	Yes (8)	No (8)	-	Yes (8)	Yes (8)
KANT R., et al.^68^	2022	IND	CA	100	Mean 50,5	M(59) F(41)	DM (80)	RO (85)	Yes (81)	MV (84)	LAMB (96)	Yes (100)	Yes (13)
							DM+H (15)	RC (11)			AmB (1)		
							NP (1)	Pulmonary (4)			POSA (1)		
							COPD (1)						
							CVA (1)						
KHICHAR S., et al.^18^	2021	IND	CC	77	Mean 46,8	M(52) F(25)	DM (56)	RSO (77)	Yes (54)	OX (31)	-	-	No (77)
MEHRABI Z., et al.^69^	2021	IRN	CA	1	51	M	-	RO	Yes	No	LAMB + CASPO	Yes	No
MEHTA S., et al.^70^	2020	IND	CA	1	60	M	DM	RO	Yes	OX/MV	LAMB	No	Yes
MEKONNEN ZK., et al.^71^	2020	USA	CA	1	60	M	DM+H+Asthma +DL	RO	Yes	OX	LAMB + POSA	Yes	Yes
MONTE JUNIOR ESD., et al.^72^	2020	BRA	CA	1	86	M	H	GI	Yes	MV	Death before treatment	No	Yes
PASERO D., et al.^16^	2021	ITA	CA	1	66	M	H+MODS	Pulmonary	No	MV	LAMB, replaced by ISA	No	Yes
PAKDEL F., et al.^73^	2021	IRN	MCS	15	Mean 52	M(10) F(5)	DM (13)	RO (15)	Yes (7)	MV (1)	LAMB (9)	Yes (15)	Yes (7)
											LAMB +POSA (3)		
											LAMB +CASPO (2)		
											LAMB +CASPO+POSA (1)		
PATEL A., et al.^74^	2021	IND	MCS	187	Mean 53,4	M(140) F(47)	DM (113)	RO (117)	Yes (48)	-	LAMB (64)	Yes (187)	Yes (145)
							HM (2)	ROC (44)			AmBD (31)		
							Trauma (3)	Pulmonary (16)			POSA (73)		
							T (3)	R (1)			ISA (19)		
								CT/GI (5)					
								D (4)					
PRADHAN P., et al.^75^	2021	IND	RE	46	Mean 48,8	M(40) F(6)	DM (44)	RO (33)	Yes (41)		LAMB (46)	Yes (46)	Yes (9)
								RC (10)					
								RS(2)					
								MA (1)					
REVANNAVAR SM., et al.^76^	2021	IND	CA	1	Middle-aged	F	DM	ROC	No	No	LAMB	Yes	No
SALDANHA M., et al.^77^	2021	IND	CA	1	32	F	DM	RO	Yes	No	LAMB	Yes	No
SELARKA L., et al.^78^	2021	IND	OS	47	Mean 55	M(35) F(12)	DM (9)	RS (12)	Yes (36)	MV (20)	LAMB (47)	Yes (47)	Yes (11)
							DM+H (27)	RO (26)		OX (18)			
							HD (6)	RC (9)		No (9)			
							COPD (2)						
							RA (1)						
							HT (2)						
SINGH SP., et al.^79^	2021	IND	SC	5	Mean 44,4	M(4) F(1)	DM (2)	ROC (5)	Yes (4)	OX (2)	LAMB (5)	Yes (5)	Yes (1)
SINGH Y., et al.^80^	2021	IND	SC	13	Mean 35	M(10) F(3)	DM (8) H (5)	RO (8)	Yes (11)	MV(10)	LAMB (11)	Yes (13)	Yes (8)
								RC (2)		OX (2)	LAMB + POSA (2)		
								RS (2)					
								Pulmonary (1)					
SINGHAI A., et al.^12^	2022	IND	CA	3	Mean 47	M(2) F(1)	DM (2)	ROC (3)	Yes (3)	OX (1)	LAMB (3)	Yes (3)	Yes (1)
SIROHIVA P., et al.^81^	2022	IND	RE	57	Median 49	M(35 F(22)	DM (42)	RS (41)	-	OX (20)	LAMB (57)	Yes (57)	Yes (7)
							H (21)	RO (14)					
							NP (14)	PA (n=2)					
VEISI A., et al.^82^	2021	IRN	CA	2	Mean 47	M(1) F(1)	DM (1)	RO (1),ROC (1)	Yes (2)	OX (2)	LAMB + Irrigation of the paranasal sinuses (2)	Yes (2)	Yes (1)

**AmB:** amphotericin B. **AmBD:** amphotericin B deoxycholate. **BRA:** Brazil. **CA:** case analysis. **CASPO:** caspofungin. **CC:** case-control. **COPD:** chronic obstructive pulmonary disease. **CS:** cross-sectional study. **CT:** cutaneous. **CVA:** cerebrovascular accident. **D:** disseminated. **DL:** dyslipidemia. **DM:** diabetes mellitus. **EGY:** Egypt. **F:** female. **GI:** gastrointestinal. **H:** hypertension. **HD:** heart disease. **HG:** hyperglycemia. **HM:** hematologic malignancy. **HT:** hyperthyroidism. **IND:** India. **IRN:** Iran. **IRQ:** Iraq. **ISA:** isavuconazole. **ITA:** Italy. **LAMB:** liposomal amphotericin B. **LD:** liver disease. **LGD:** lung disease. **M:** male. **MA:** mandibular. **MCS:** multicenter study. **MODS:** multiple organ dysfunction syndrome. **MS:** myelodysplastic syndrome. **MV:** mechanical ventilation. **MX:** maxillary. **MYS:** Malaysia. **N:** number of cases. **NP:** nephropathy. **OD:** other diseases. **OR:** orbital. **OS:** observational study. **OX:** oxygen therapy. **PA:** palatal. **POSA:** posaconazole. **R:** renal. **RA:** rheumatoid arthritis. **RC:** rhino-cerebral. **RE:** retrospective study. **RO:** rhino-orbital. **ROC:** rhino-orbito-cerebral. **RS:** rhino-sinus. **RSO:** rhino-sino-orbital. **S:** subglottic. **SC:** case series. **SI:** surgical intervention. **T:** transplant. **TUR:** Turkey. **USA:** United States of America.

## DISCUSSION

As the COVID-19 pandemic progressed, an increasing number of patients under treatment began to present with mucormycosis, a severe invasive fungal infection^22^. Several studies have since documented a high rate of COVID-19/mucormycosis co-infection, with approximately 80% of cases occurring in Indian patients^15^ ^,^ ^23^. The present review synthesis data from 1,482 published cases, providing a comprehensive overview of epidemiological, clinical, and therapeutic aspects. However, these findings are based on descriptive aggregation of heterogeneous data sources and should be interpreted with caution, as they may reflect reporting patterns rather than true epidemiological distributions.

Mucorales fungi are transmitted primarily by inhalation of spores, ingestion of contaminated food, or traumatic inoculation^24^. The route of transmission influences the site of fungal infection, which can present as rhino-cerebral, rhino-orbital, rhino-sinus, rhino-orbitocerebral, pulmonary, gastrointestinal, cutaneous or disseminated forms^25^.

Exposure of immunocompetent individuals to fungal spores usually does not cause disease due to the effectiveness of the host immune system^13^. *Mucor* spp. and *Rhizopus* spp. are opportunistic pathogens that require a compromised immune system to establish infection, a condition frequently observed in patients with severe COVID-19^26^.

The development of mucormycosis requires fungi to evade host immune responses, adhere to the endothelium, undergo endocytosis, form hyphae, cause endothelial damage, and disseminate via the bloodstream. This scenario characterizes mucormycosis as an angioinvasive disease with a strong tendency to invade blood vessels, causing ischemia and necrosis^27^. This vascular tropism is related to the ability of fungi to adhere to endothelial cells, a critical step in the establishment of infection. Another virulence factor is the ability of pathogens to scavenge iron from the host, which is essential for fungal germination^28^. After inoculation, fungal spores bind to extracellular matrix proteins and secrete enzymes that destroy host tissues, contributing to their ischemic and necrotic potential^17^.

Iron is essential for fungal growth and replication. Mucorales fungi extract iron from their host. In healthy individuals, iron is bound to serum proteins. However, in patients with uncontrolled diabetes, free serum iron levels increase, resulting in fungal growth and alterations in phagocytic activity^25^ ^,^ ^29^.

The primary innate immune defense against fungal spores includes circulating neutrophils, mononuclear cells, and macrophages. Tissue and alveolar macrophages ingest spores and destroy them. If hyphae survive, neutrophil chemotaxis is activated, leading to the release of reactive oxygen species and pro-inflammatory cytokines, which recruit and activate other immune cells^30^. However, patients with COVID-19 may experience cytokine storms, and corticosteroid treatment leads to immunosuppression, making the host less capable of controlling fungal proliferation^31^.

Mucormycosis can affect various anatomical sites. The literature has documented rhino-orbital, rhino-sinus, rhino-orbito-cerebral, pulmonary, and disseminated forms, among others. Most patients with COVID-19/mucormycosis co-infection presented rhino-orbital involvement, followed by rhino-sinus and rhino-orbito-cerebral forms. Pulmonary, cutaneous, and maxillary forms were reported less frequently^32^. These findings are consistent with those of the present review.

Patients with COVID-19/mucormycosis co-infection may present with various clinical manifestations. Edema (ocular or facial) has been reported in up to 61% of cases, and ptosis in 54%. Other frequent symptoms included proptosis, headache, and facial and ocular pain^33^ ^,^ ^34^. Some studies have also identified headache, fever, facial numbness or pain, and erythema around the eyes or nose as the most common manifestations^35^. The most frequently observed symptoms in this review were fever, headache, ptosis, proptosis, ophthalmoplegia, and edema, which correspond to the most prevalent clinical forms identified.

A retrospective study of 67 patients with COVID-19/mucormycosis identified risk factors including diabetes mellitus, hyperglycemia, and corticosteroid use for controlling SARS-CoV-2-induced inflammation^36^. Other studies found diabetes in more than 78% of cases, hypertension in 80%, and renal failure in 10%^33^ ^,^ ^37^ ^,^ ^38^. Comorbidities such as chronic obstructive pulmonary disease (COPD), renal disease, and liver disease also appeared in up to 22% of cases^37^.

The use of corticosteroids during COVID-19 treatment is another primary risk factor. Prolonged corticosteroids use causes immunosuppression and hyperglycemia by inducing excessive glycosylation of proteins, such as transferrin and ferritin, thus reducing their iron-binding affinity and favoring fungal proliferation^39^. A systematic review involving 3,354 patients found that 71% had diabetes mellitus and 78% received corticosteroids as part of COVID-19 treatment^38^. High doses of corticosteroids not only suppress the immune system but also disrupt glycemic control^40^. In the present review, 64.3% (n = 953) of patients received corticosteroids.

Oxygen therapy and mechanical ventilation, often required in cases of severe COVID-19, also represent risk factors. Sen et al. (2021) reported that 72% of hospitalized COVID-19 patients with mucormycosis received some form of oxygen support. A study conducted in Mumbai employed oxygen therapy in 11% and mechanical ventilation in 0.2% of cases^41^. In the present review, oxygen therapy was necessary in 23.7% of patients, and mechanical ventilation in 9.4%.

Regarding treatment, the Delphi consensus of the Fungal Infection Study Forum and the Academy of Pulmonary Sciences of India recommended liposomal amphotericin B along with surgical intervention when necessary^42^. Liposomal amphotericin B is the gold standard due to its lower nephrotoxicity and suitability for prolonged use than the deoxycholate form^43^ ^,^ ^44^. Isavuconazole and posaconazole are also effective but have slower bioavailability. Although not commonly recommended due to high toxicity, deoxycholate amphotericin B is used in resource-limited settings such as India^45^. Itraconazole can be considered in the absence of other options, although it is not first-line therapy^44^. In most studies, liposomal amphotericin B was the primary treatment. Despite appropriate antifungal therapy, surgical intervention was required in about 70% of cases^34^ ^,^ ^46^.

In the present review, 49.5% of patients received amphotericin B (any formulation), 4.6% received combination therapy, and 55.5% underwent surgery. The mortality rate was 25%, while clinical recovery occurred in 64.8% of cases.

The marked predominance of cases from India (>90%) must be interpreted in the context of regional factors, including a high prevalence of uncontrolled diabetes, widespread corticosteroid use, environmental exposure to fungal spores, and more intensive surveillance and reporting. Therefore, the findings of this review may not fully represent the global epidemiology of COVID-19-associated mucormycosis.

Although a decline in reported cases has been observed in the post-vaccination period, the clinical and epidemiological insights gained during the COVID-19 pandemic remain highly relevant. Mucormycosis continues to pose a significant threat to vulnerable populations, particularly in settings with a high burden of comorbidities such as diabetes mellitus and immunosuppression. Furthermore, the challenges related to early diagnosis and timely treatment persist beyond the pandemic period.

In this context, the present study contributes to consolidating available evidence in Brazil and highlights key aspects that may inform clinical management and guide future research, including in post-pandemic scenarios.

## CONCLUSION

COVID-19-associated mucormycosis emerged as a significant complication during the acute phases of the pandemic, particularly in settings with a high prevalence of diabetes and widespread use of corticosteroids. This review of 1,482 published cases highlights the central role of metabolic dysregulation and immunosuppression as key risk factors. Although a marked decline in reported cases has been observed after 2022, this trend is based on heterogeneous data sources, including published studies and surveillance reports, and should be interpreted with caution.

The apparent reduction in incidence may be associated with multiple factors, such as improved clinical management, more judicious use of corticosteroids, better glycemic control, and widespread vaccination; however, causal relationships cannot be definitively established. These findings reinforce the importance of careful metabolic and immunological management in critically ill patients, particularly in the context of future infectious disease outbreaks.

## Data Availability

Data-in-article: Research data is available in the body of the document (Table 2).
