# Supplementary material for: COVID-19-associated mucormycosis: the rise and fall of an epidemic within a pandemic - a systematic review of 1,482 cases (2019-2025)
Source: Rev Soc Bras Med Trop. 2026 Jul 17;59:e0504-2025. doi: 10.1590/0037-8682-0504-2025 (PMC13379234; doi:10.1590/0037-8682-0504-2025)
Supplement: Supplementary material [file 1678-9849-rsbmt-59-e0504-2025-md1.pdf]

TABLE S1: Search strategy for each database.

Database	Complete search strategy
PubMed	("COVID-19-associated mucormycosis"[Title/Abstract] OR "SARS-CoV-2"[Title/Abstract] OR "COVID-19"[Title/Abstract]) AND (mucormycosis[Title/Abstract] OR *Mucor*[Title/Abstract] OR *Rhizopus*[Title/Abstract])
Embase	('covid-19-associated mucormycosis':ti,ab OR 'sars-cov-2':ti,ab OR 'covid-19':ti,ab) AND (mucormycosis:ti,ab OR *mucor*:ti,ab OR *rhizopus*:ti,ab)
Scielo	("COVID-19-associated mucormycosis" OR "SARS-CoV-2" OR "COVID-19") AND ("mucormycosis" OR *Mucor* OR *Rhizopus*)
