# Supplementary material for: COVID-19-associated mucormycosis: the rise and fall of an epidemic within a pandemic - a systematic review of 1,482 cases (2019-2025)
Source: Rev Soc Bras Med Trop. 2026 Jul 17;59:e0504-2025. doi: 10.1590/0037-8682-0504-2025 (PMC13379234; doi:10.1590/0037-8682-0504-2025)
Supplement: Supplementary material [file 1678-9849-rsbmt-59-e0504-2025-md2.pdf]

TABLE S2. Methodological quality assessment of included studies using the Joanna Briggs Institute (JBI) Critical Appraisal Checklists, stratified according to study design (case reports).

Case Reports	Q1	Q2	Q3	Q4	Q5	Q6	Q7	Q8
Alekseyev et al., 2021	Y	Y	Y	Y	Y	Y	Y	Y
Amirzargar et al., 2022	Y	Y	Y	Y	Y	Y	Y	Y
Chang et al., 2022	Y	Y	Y	Y	Y	Y	Y	Y
Eswaran et al., 2021	Y	Y	Y	Y	Y	Y	Y	Y
Mehrabi et al., 2021	Y	Y	Y	Y	Y	Y	Y	Y
Mehta et al., 2020	Y	Y	Y	Y	Y	Y	Y	Y
Mekonnen et al., 2020	Y	Y	Y	Y	Y	Y	Y	Y
Monte Junior et al., 2020	Y	Y	Y	Y	Y	Y	Y	Y
Pasero et al., 2021	Y	Y	Y	Y	Y	Y	Y	N
Revannavar et al., 2021	Y	Y	Y	Y	Y	Y	Y	Y
Saldanha et al., 2021	Y	Y	Y	Y	Y	Y	Y	Y
Singhai et al., 2022	Y	Y	Y	Y	Y	Y	Y	Y
Veisi et al., 2021	Y	Y	Y	Y	Y	Y	Y	Y

Q1: Patient demographic characteristics clearly described; Q2: Patient history clearly described and presented as a timeline; Q3: Clinical condition on presentation clearly described; Q4: Diagnostic tests and results clearly described; Q5: Intervention(s) or treatment procedure(s) clearly described; Q6: Post-intervention clinical condition clearly described; Q7: Adverse events (or unanticipated events) reported; Q8: Takeaway lessons provided; Y: Yes; N: No.
