# Supplementary material for: COVID-19-associated mucormycosis: the rise and fall of an epidemic within a pandemic - a systematic review of 1,482 cases (2019-2025)
Source: Rev Soc Bras Med Trop. 2026 Jul 17;59:e0504-2025. doi: 10.1590/0037-8682-0504-2025 (PMC13379234; doi:10.1590/0037-8682-0504-2025)
Supplement: Supplementary material [file 1678-9849-rsbmt-59-e0504-2025-md3.pdf]

TABLE S3. Methodological quality assessment of included studies using the Joanna Briggs Institute (JBI) Critical Appraisal Checklists, stratified according to study design (case series).

Case Series	Q1	Q2	Q3	Q4	Q5	Q6	Q7	Q8	Q9	Q10
Aggarwal et al., 2021	Y	Y	Y	Y	Y	Y	Y	Y	Y	Y
Ahmed et al., 2022	Y	Y	Y	Y	Y	Y	Y	Y	N	Y
Alloush et al., 2022	Y	Y	Y	Y	Y	Y	Y	Y	N	Y
Arora R. et al., 2021	Y	Y	Y	Y	Y	Y	Y	Y	N	Y
Arora U. et al., 2022	Y	Y	Y	Y	Y	Y	Y	Y	N	Y
Avatef Fazeli et al., 2021	Y	Y	Y	Y	Y	Y	Y	Y	Y	Y
Barman Roy et al., 2021	Y	Y	Y	Y	Y	Y	Y	Y	N	Y
Bayram et al., 2021	Y	Y	Y	Y	Y	Y	Y	Y	N	Y
Bhanuprasad et al., 2021	Y	Y	Y	Y	Y	Y	Y	Y	N	Y
Chakravarty et al., 2022	Y	Y	Y	Y	Y	Y	Y	Y	N	Y
Dravid et al., 2022	Y	Y	Y	Y	Y	Y	Y	Y	N	Y
Farghly et al., 2022	Y	Y	Y	Y	Y	Y	Y	Y	N	Y
Fouad et al., 2021a	Y	Y	Y	Y	Y	Y	Y	Y	Y	Y
Fouad et al., 2021b	Y	Y	Y	Y	Y	Y	Y	Y	N	Y
Gupta et al., 2021	Y	Y	Y	Y	Y	Y	Y	Y	N	Y
Hasheminasab et al., 2022	Y	Y	Y	Y	Y	Y	Y	Y	Y	Y
Kant et al., 2022	Y	Y	Y	Y	Y	Y	Y	Y	N	Y
Khichar et al., 2021	Y	Y	Y	Y	Y	Y	Y	Y	N	Y
Pakdel et al., 2021	Y	Y	Y	Y	Y	Y	Y	Y	N	Y
Patel et al., 2021	Y	Y	Y	Y	Y	Y	Y	Y	N	Y
Pradhan et al., 2021	Y	Y	Y	Y	Y	Y	Y	Y	N	Y
Selarka et al., 2021	Y	Y	Y	Y	Y	Y	Y	Y	N	Y
Singh SP et al., 2021	Y	Y	Y	Y	Y	Y	Y	Y	Y	Y
Singh Y et al., 2021	Y	Y	Y	Y	Y	Y	Y	Y	N	Y
Sirohiya et al., 2022	Y	Y	Y	Y	Y	Y	Y	Y	N	Y

Q1: Clear criteria for inclusion; Q2: Condition measured in a standard, reliable way; Q3: Valid methods used for condition identification; Q4: Consecutive inclusion of participants; Q5: Complete inclusion of participants; Q6: Clear reporting of participant demographics; Q7: Clear reporting of clinical information; Q8: Outcomes or follow-up results clearly reported; Q9: Clear reporting of presenting sites/clinical settings; Q10: Appropriate statistical analysis; Y: Yes; N: No.
